# Effect of metatarsal osteotomy and open lateral soft tissue procedure on sesamoid position: radiological assessment

**DOI:** 10.1186/s13018-017-0712-y

**Published:** 2018-01-16

**Authors:** Young Rak Choi, Sang-June Lee, Jun Hyun Kim, Tae Ho Kim, Chi Hoon Oh

**Affiliations:** Department of Orthopaedic Surgery, CHA Bundang Medical Center, School of Medicine, CHA University, Seong-nam, Republic of Korea

**Keywords:** Hallux valgus, Chevron osteotomy, Open lateral soft tissue procedure, Sesamoid position

## Abstract

**Background:**

Incomplete sesamoid reduction is a potential risk factor for the recurrence of hallux valgus. The purpose of this study was to radiologically investigate changes in sesamoid position after chevron osteotomy and the open lateral soft tissue procedure.

**Methods:**

Sixty-eight feet that underwent operative correction for hallux valgus deformity were reviewed consecutively. The hallux valgus angle (HVA), first to second intermetatarsal angle (IMA), tibial sesamoid position (TSP), distance of the fibular sesamoid (DFS), and translation of the metatarsal head (TMH) were evaluated preoperatively and at final follow-up.

**Results:**

While most parameters were significantly decreased after surgery, no significant change in DFS (correction − 1.45 mm, *p* = 0.08) was noted. The difference between preoperative and postoperative TSP values (ΔTSP) has a moderately positive correlation with difference in TMH values (ΔTMH) (Rho 0.475, *p* = .000). Other parameters were similarly correlated.

**Conclusions:**

First, metatarsal bone realignment reduced the sesamoid, but its position, relative to the second metatarsal axis (DFS), was unchanged. The sesamoid is reduced by the lateral translation of the first metatarsal but not by medial sesamoid migration.

## Background

Hallux valgus deformity, a common forefoot disease, is characterized by lateral subluxation of the first metatarsophalangeal joint, metatarsus primus varus, and lateral rotation of the sesamoids. The prevalence of hallux valgus is estimated to be over 23% in adults and up to 35.7% in the elderly [1]. The majority of corrective procedures for hallux valgus use a combination of soft tissue procedure and osteotomy [2–11]. Correcting sesamoid subluxation is an important component of hallux valgus reconstruction, as it removes the dynamic deforming force of the flexor hallux brevis muscle. Failure to correct sesamoid subluxation can lead to a recurrence of hallux valgus, sesamoid overload or irritation, and altered walking mechanics [12]. The degree of sesamoid lateral displacement is highly correlated with the severity of hallux valgus deformity. In previous study, many researchers found a correlation between incomplete sesamoid reduction and radiographic evidence of recurrent hallux valgus. These findings suggest that following operative correction of hallux valgus deformity, intraoperative confirmation that the sesamoids are under the first metatarsal head is necessary [13]. Here, we analyzed the radiographic effects of chevron osteotomy with an open lateral soft tissue procedure on sesamoid position.

## Methods

Sixty-eight feet from 52 patients that underwent operative correction of hallux valgus deformity from 2010 to 2016 by one surgeon were reviewed retrospectively. Symptomatic moderate to severe hallux valgus deformities, defined by a hallux valgus angle ≥ 25° with subluxation of the first metatarsophalangeal joint or an intermetatarsal angle ≥ 12°, were treated with an open lateral soft tissue procedure combined with a chevron osteotomy of the first metatarsal. Patients with a history of previous foot surgeries, revision corrections, or other inflammatory diseases or who were lost to follow-up were excluded from this study. Anterior-posterior weight-bearing radiographs of the feet were taken preoperatively and at the time of final follow-up.

### Operative technique

**Fig. 1:**
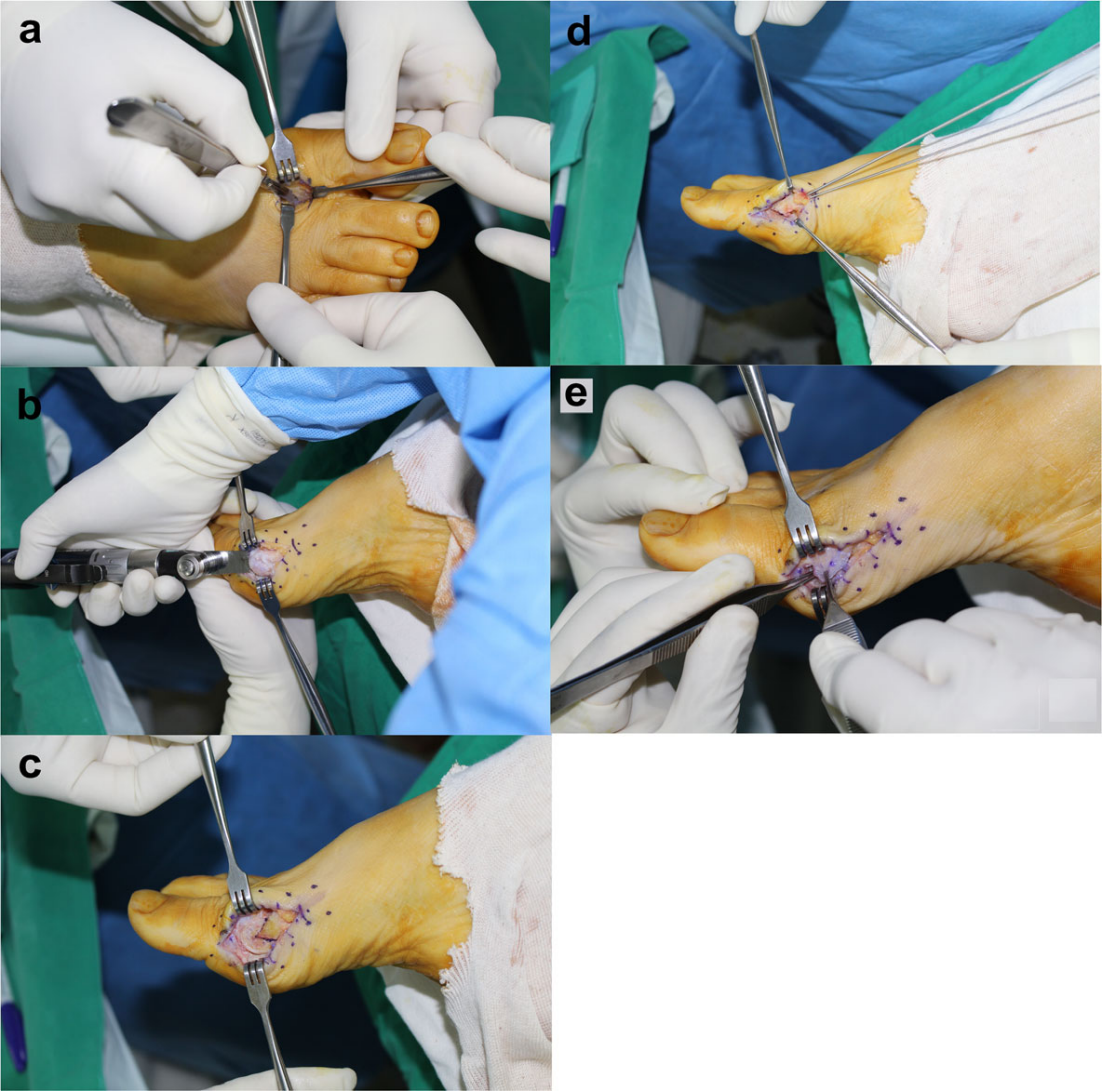
Operative technique. **a** Complete release of the adductor hallucis tendon and the metatarso-sesamoidal ligament. **b** Excision of the medial eminence. **c** Distal chevron metatarsal osteotomy. **d** K-wire fixation. **e** Capsular plication

A distal or proximal chevron osteotomy with an open lateral soft tissue procedure was performed. A T-shaped medial approach to the first metatarsophalangeal joint was typically followed by a 2- to 3- mm excision of the medial eminence with a sagittal saw and a concomitant, complete release of the adductor hallucis tendon and metatarso-sesamoidal ligament. After the open lateral soft tissue procedure, a chevron osteotomy was conducted in the sagittal plane using a sharp saw blade. The metatarsal fragment was subsequently translated 5- to 7- mm laterally for deformity correction. For stability, 2- or 3 0.062-mm K-wires were placed. Finally, medial capsular plication was performed (Fig. 1).

### Radiographic measurements

Weight bearing anterior-posterior radiographs were analyzed preoperatively and at final follow-up. All radiologic data were assessed by two orthopedic surgeons who were not involved in these surgeries.

#### Distance of fibular sesamoid (DFS)

**Fig. 2:**
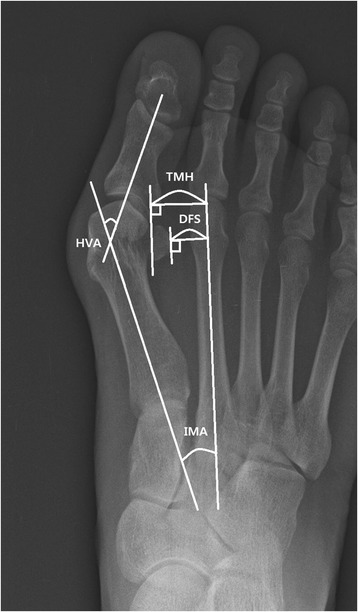
Measurement of IMA, HVA, DFS, and TMH

Fibular sesamoid position was analyzed by measuring the perpendicular distance from the second metatarsal axis to the lateral margin of the fibular sesamoid (Fig. 2).

#### Tibial sesamoid position (TSP)

**Fig. 3:**
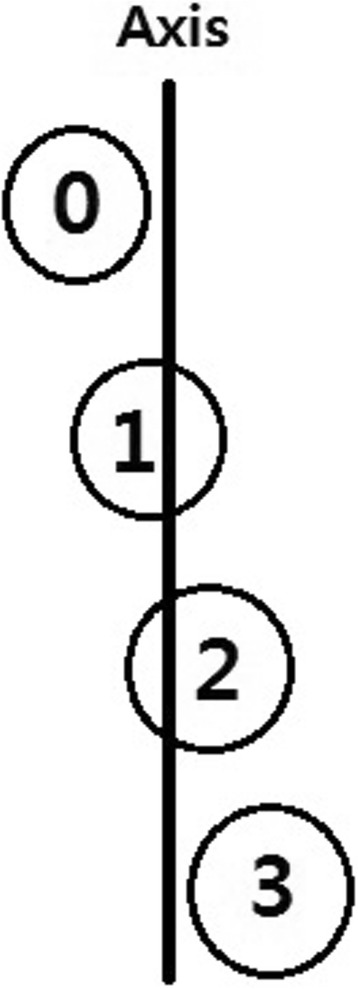
Measurement of TSP

Tibial sesamoid position was measured using the American Orthopedic Foot Society grading scale, which consists of four position assessments. This method relates the position of the tibial sesamoid to the longitudinal axis of the first metatarsal. A sesamoid with no lateral displacement relative to a line bisecting this axis is defined as grade 0; an overlap of < 50%, as grade 1; an overlap of > 50%, as grade 2; and complete lateral displacement beyond the reference line, as grade 3 (Fig. 3) [14].

#### Hallux valgus angle (HVA), first to second intermetatarsal angle (IMA)

The hallux valgus angle (HVA) is a measure of the angle created by the long axis of the first metatarsal and the proximal phalanx. The first to second intermetatarsal angle (IMA) is the angle created by the long axis of the first and second metatarsals (Fig. 2).

#### Translation of first distal metatarsal head (TMH)

Translation of the first distal metatarsal head was assessed by measuring the perpendicular distance between the second metatarsal axis and the distal metatarsal head lateral articulation (Fig. 2).

### Statistical analysis

A paired *t*-test was used to evaluate differences between measurements taken preoperatively and at final follow-up. *P* values < 0.05 were defined as significant. Reliability analysis was used to analyze inter-observer correlation. Simple correlation analysis was used to assess correlations between ΔHVA, ΔIMA, ΔDFS, ΔTSP, and ΔTMH. The data were analyzed using SPSS, version 21.0 statistical software package (IBM Corp., Armonk, NY).

## Results

Three patients were male, and 49 were female. Twenty-three of 68 feet underwent proximal chevron osteotomy and 45 of 68 feet underwent distal chevron osteotomy. The mean age of the patients at the time of surgery was 52 (range, 18–79) years. Both feet were affected in 19 patients, while unilateral disease was noted in 30 patients. The mean follow-up was 18 (range, 12–30) months (Table 1).

**Table 1:** Preoperative and postoperative measurements of hallux valgus

	Preoperative (*n* = 75)	Postoperative (*n* = 75)	Correction	*P* value
HVA	32.40	7.56	− 24.84	< .001
IMA	15.36	4.81	− 10.55	< .001
TSP	2.56	1.35	− 1.21	< .001
DFS	16.03	14.54	− 1.49	0.08
TMH	19.01	15.29	− 3.72	< .001

Values are expressed as the mean (standard deviation)

*Abbreviations*: *HVA* Hallux valgus angle, *IMA* intermetatarsal angle, *TSP* ttibial sesamoid position, *DFS* distance of the fibular sesamoid bone from the second metatarsal axis, *TMA* translation of the first distal metatarsal head

Postoperative HVA, IMA, TSP and TMH were significantly decreased. The average changes in HVA were from 32.40° preoperatively to 7.56° (*p* < .001) at final follow-up, and IMA decreased from 15.36° to 4.81°. The median TSP decreased from 2.56 preoperatively to 1.35 at final follow-up (*p* < .001). TMH decreased from 19.01 to 15.29 mm, and DFS decreased from 16.03 to 14.54 mm (*p* = 0.08) (Table 2).

**Table 2:** Pearson correlation coefficients between ΔHVA, ΔIMA, ΔTSP, ΔDFS, and ΔTMH

		ΔHVA	ΔIMA	ΔTSP	ΔDFS	ΔTMH
ΔHVA	Rho					
	*P* value					
ΔIMA	Rho	0.475				
	*P* value	0.000^**^				
ΔTSP	Rho	0.091	0.275			
	*P* value	0.459	0.023^*^			
ΔDFS	Rho	0.355	0.377	0.089		
	*P* value	0.003^**^	0.002^**^	0.470		
ΔTMH	Rho	0.388	0.443	0.475	0.452	
	*P* value	0.001^**^	0.000^**^	0.000^**^	0.000^**^	

ΔDifference between preoperative and postoperative values

*P value < 0.05; **P value < 0.01

*Abbreviations*: Rho, Pearson correlation coefficient

The Pearson correlation coefficients between ΔHVA, ΔIMA, ΔTSP, ΔDFS, and ΔTMH show a weak to moderate positive correlation. The highest positive correlation was between ΔTSP and ΔTMH (*p* = .000, *r* = 0.475). In contrast, significant correlations were not observed for ΔHVA with ΔTSP or ΔDFS with ΔTSP.

## Discussion

A change in sesamoid position has been recognized as a risk factor for hallux valgus recurrence [13]. Mitchell et al. reported that a loss of alignment between the first metatarsal head and the sesamoid causes metatarsalgia [15]. Correction of the metatarso-phalangeal-sesamoid joint complex reduced the risk of hallux valgus recurrence [14].

The surgical techniques for sesamoid correction have been well-investigated because of the importance of sesamoid position. Distal soft tissue procedures reportedly have good outcomes and are still performed by many surgeons. Bai et al. reported that a distal chevron osteotomy with a distal soft tissue procedure is an effective and reliable means of correcting moderate to severe hallux valgus deformity [16]. Park et al. reported that a distal chevron osteotomy with an associated distal soft-tissue procedure is satisfactory for correcting severe hallux valgus deformities [17]. Pochatko et al. reported good outcomes following a distal chevron osteotomy combined with a lateral release [18]. Although these studies did not focus on distal soft tissue procedures and differed in methodology, many surgeons agree on the effectiveness of soft tissue procedures.

In contrast, many reports indicate that sesamoid position is reduced by a first metatarsal osteotomy; in addition, a soft tissue procedure alone is inadequate for reducing sesamoid subluxation because the sesamoid and first metatarsal move independently. Huang et al. suggested that the majority of the sesamoid correction correlates with first to second intermetatarsal correction. Sesamoid reduction beneath the first metatarsal is due to bony realignment of the first metatarsal over the sesamoids [12]. Woo et al. reported that despite significantly improved clinical and radiologic outcomes after bony realignment with lateral soft tissue release, this procedure does not result in medial sesamoid shift or a reduction in sesamoid position [19]. Geng et al. reported that the sesamoids retaining their relationship with the second metatarsal and medial drifting of the first metatarsal but not with the lateral migration of the sesamoids is what heads to the subluxation between the first metatarsal and sesamoids [20].

In our study, there was no significant change in DFS after surgery. A minimal decrease in DFS was predicted because of the metatarso-phalangeo-sesamoidal linkage. We presumed that the sesamoid would be shifted laterally, corresponding to lateral translation of the first distal metatarsal head fragment. Even if sesamoid reduction following an open lateral soft tissue procedure was offset by distal metatarsal fragment translation, the difference would be too minimal. We therefore could not show any effect of open lateral soft tissue procedure that would alter intrinsic position of sesamoid bone. A significant moderately positive correlation between TMH and TSP was confirmed. We therefore confirmed that proper realignment of the first metatarsal is the most important sesamoid reduction method. Further, the relative distance from the lateral sesamoid to the second metatarsal axis does not change following chevron osteotomy with an open lateral soft tissue procedure. The intrinsic position of the sesamoid bone was not changed following surgical correction.

This study had some limitations. First, we defined sesamoid position as the absolute position in a two-dimensional plane. However, true sesamoid position appears to be more complicated and thus must be considered a rotational change. Second, the exact mechanism of the change in DFS is unknown, but this change is likely due to the connection between the sesamoid bone and the first distal metatarsal fragment. A cadaveric study is needed to assess anatomic changes. Finally, a randomized control study is needed to compare soft tissue and non-soft tissue procedures.

## Conclusions

In conclusion, first metatarsal realignment reduced the position of the sesamoid, but its intrinsic position relative to the second metatarsal axis is unchanged. The sesamoid is reduced by lateral translation of the first metatarsal but not by medial migration of the sesamoids.

## Abbreviations

DFS: Distance of the fibular sesamoid bone from the second metatarsal axis
HVA: Hallux valgus angle
IMA: Intermetatarsal angle
TMA: Translation of the first distal metatarsal head
TSP: Tibial sesamoid position
